# Study on the factors affecting cracking of earthen soil under dry shrinkage and freeze–thaw conditions

**DOI:** 10.1038/s41598-022-05946-w

**Published:** 2022-02-02

**Authors:** Jianwei Yue, Xuanjia Huang, Limin Zhao, Zifa Wang

**Affiliations:** 1 School of Civil Engineering and Architecture, Henan University, Kaifeng, 475004 China; 2 Institute of Safety Evaluation and Restoration of Immovable Cultural Relics, Henan University, Kaifeng, 475004 China; 3 Henan Universityniversity Yellow River Civilization and Sustainable Development Research Center, Kaifeng, 475004 China

**Keywords:** Civil engineering, Environmental impact

## Abstract

Earthen sites are easily eroded by the natural environment, resulting in many micro-cracks on the surface. To explore the effects of environmental effects such as drying shrinkage and freeze–thaw on surface cracking, orthogonal tests that imposed these effects on the Zhouqiao site were conducted. Using range analysis, image processing technology, surface strength measurement and microstructure characteristic analysis, this paper explores the effects of soil thickness, water content, dry shrinkage, freeze–thaw cycles and other factors on the morphological characteristics of the site’s surface cracks. The results show that under the action of dry shrinkage, the thickness of soil layer is the primary factor affecting the cracking of earthen soil. The thinner the thickness of soil layer, the lower the moisture content, and the more serious the cracking. The initial moisture content is the most disadvantageous factor affecting the reduction of the surface strength of the earthen soil. The strength around the soil sample is lower than that inside, and there are more cracks. Under the action of freezing and thawing, the main factors affecting the cracking and surface strength reduction of earthen soil are the initial water content and soil layer thickness, and the thicker the soil layer, the smaller the crack development and the lower the surface strength. Scanning electron microscope results show that under dry shrinkage and freeze–thaw conditions, the internal cracks of the soil samples exhibit different shape characteristics. Intergranular cracks appear most often under dry shrinkage conditions, and isolated cracks appear most often in the soil samples from the freeze–thaw cycle test. The cracks caused by these two types of external environment factors damage the earthen soil. According to the tension failure model and the definition of the first frost heaving theory, it can be determined that when the micro pore force *F* and the maximum frost heaving pressure *P*_*Imax*_ are greater than the cohesion of the soil sample, the soil sample will germinate cracks.

## Introduction

**Figure 1:**
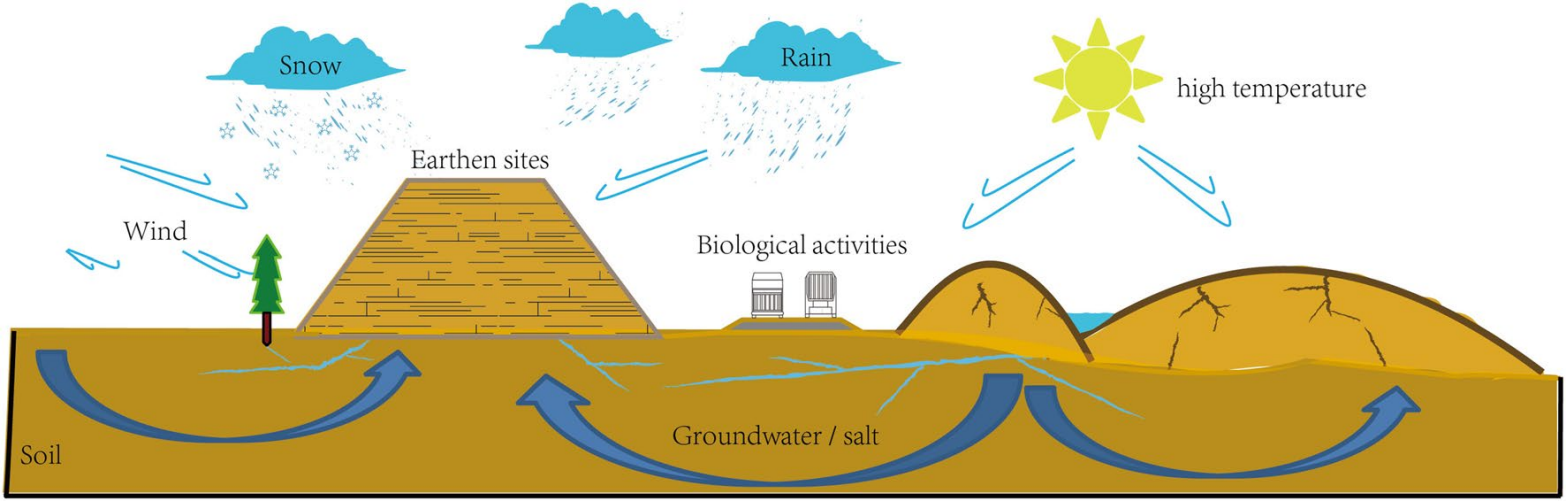
Schematic diagram of multi factor action of earthen sites.

Earthen sites are affected by the natural environment, man-made destruction and other factors, and most earthen sites are facing serious threats^1,2^. The arid and rainless environment in the Central Plains is quite different from that in the northwest^3^. The arid and rainless environment in the northwest is conducive to the protection of soil sites^4^, while the rainy summer and cold winter climate in the Central Plains accelerate the deterioration of earthen sites^5–7^ (Fig. 1), which makes it more difficult to protect earthen sites^8^. However, the complexity, diversity and particularity of the deterioration mechanisms of earthen sites in the Central Plains^9^ highlight the necessity and urgency of preventive protection^10^.

Scholars at home and abroad have contributed certain research results regarding the mechanical properties and deterioration characteristics of geotechnical materials^11–15^; many found that soil sites are vulnerable to erosion by the natural environment, and a large number of cracks generally develop on the surface, where dry shrinkage and freeze-thaw are common influencing factors^16,17^. Much of the current research has focused on the macro-microscopic changes in the development of fractures in soil samples under dry shrinkage and freeze-thaw conditions. Earthen site cracking is related to external environmental effects and microstructural composition. Under the action of the external environment, the soil loses water and shrinks internally, and is subjected to boundary constraints that generate tensile stresses internally, and the microstructure changes. When the tensile stresses exceed the tensile strength of the soil itself, the physical and mechanical properties of the soil sample gradually weaken, and eventually cracks are formed^18,19^. Under the conditions of dry shrinkage and freeze-thaw, the interaction caused by water migration in the soil breaks the micro particle skeleton of the soil sample, reduces the strength of the soil sample, and leads to the destruction of the earthen site^20,21^. The cracking of soil samples under freeze-thaw and dry wet action is analyzed by means of the combination of macro and micro. It can be found that the freeze-thaw cycle mainly produces invisible micro cracks in the soil sample, and the dry wet cycle mainly produces visible macro cracks. These changes are related to the water content of the soil sample^22,23^. Shi et al.^24^ found that the times of freezing and thawing will affect the strength characteristics of soil samples, and pointed out that erosion destroys the cementation between soil particles, changes the arrangement structure between soil particles, and weakens the mechanical strength of soil. Zhao et al.^25^ found in the dry shrinkage and freeze-thaw cycle test that the moisture content greatly affects the development of microcracks and the composition of microstructure of clay, resulting in the reduction of the mechanical properties of soil samples.

It can be seen from the above analysis that under dry shrinkage and freeze-thaw conditions, the research method used to elucidate the engineering characteristics of earthen soil is relatively singular^26^, and the effects of factors such as temperature, humidity, soil sample thickness, dry shrinkage and freeze-thaw cycles on the morphological characteristics of fracture networks are not considered. Some research results have been contributed by studying soil sites in the dry areas of Western China, but there is little data regarding the ontological safety and stability of soil sites under different conditions in the central plains^27^. The soils in this region do not match the number and value of the soil sites found in the Central Plains. In this paper, through dry shrinkage and freeze-thaw orthogonal tests on the Zhouqiao site’s earthen soil using range analysis, image processing technology, surface strength measurement and microstructure analysis, we comprehensively explore the effects of influencing factors such as soil thickness, moisture content, dry-shrinkage and freeze-thaw cycles on the morphological characteristics of earthen soil fissure networks.

## Methods

### Basic properties of earthen soil samples

The test soils were taken from the upper, middle and lower sections of the Zhouqiao site in Kaifeng City. The returned soil samples were tested according to the standard for geotechnical test methods (GB/T50123-2019). The basic physical indexes of the soil samples are shown in Table 1. Based on a comprehensive analysis of the geotechnical test results, it was found that the water content distribution of the soil samples in the upper, middle and lower layers is uneven. After measurement, the water content of the soil samples in the upper section of the site is low and the surface crack depth is deep. The soil samples at the site’s lower inner corner have high moisture content and shallow surface crack depth. Therefore, soil thickness is an important factor considered in this paper.

**Table 1:** Basic properties of soil samples from Zhouqiao site.

Soil sample	Liquid limit/%	Plastic limit/%	Plasticity index	Natural dry density/(g/cm^3^)	Natural moisture content/%
Upper soil	35.5	21.2	14.3	1.63	12.5
Middle soil	32.8	19.7	13.1	1.65	14.3
Under soil	30.9	18.5	12.4	1.67	15.6

### Orthogonal test

**Figure 2:**
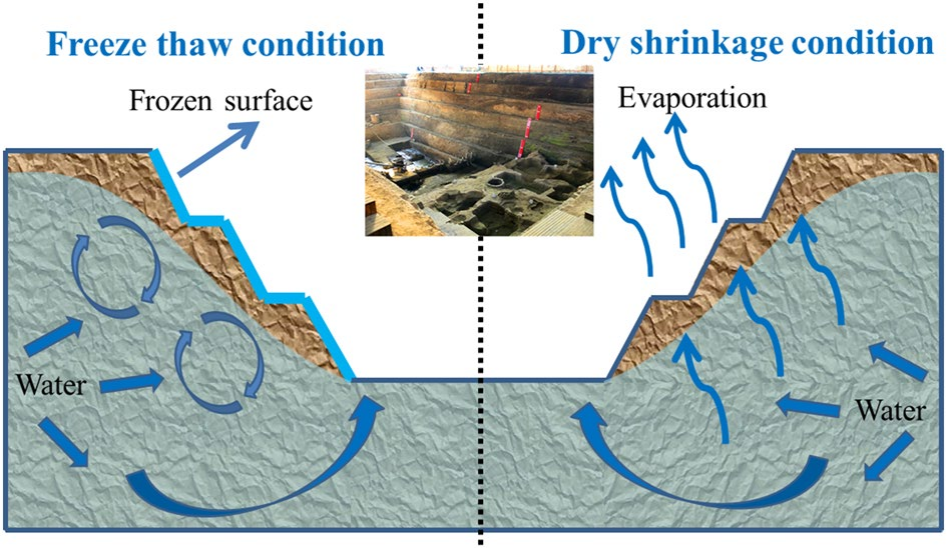
Schematic diagram of working conditions of Zhouqiao site under dry shrinkage and freeze–thaw conditions.

Through a field survey of the Zhouqiao site, it was found that there is a water pit in the middle of the site’s test pit and that the water around the site is constantly seeping under pressure. The migration of water has a certain impact on the stability of the site itself^28^ (Fig. 2). Under the dry shrinkage condition, the water in the earthen site continuously migrates to the surface and evaporates into the atmosphere. Under the conditions of freezing and thawing, the earthen site’s surface water crystallizes in the intergranular pores^29^, forming a frozen surface. When the water migrates to a certain extent, it forms ice rimming. When it melts, the intergranular pores are expanded by crystallization shrink, resulting in microcracking of the site soil. After multiple freezing and thawing cycles, a crack network is finally formed. Therefore, a method for determining the influencing factors of site soil cracking under dry shrinkage and freeze-thaw conditions is very important.

#### Dry shrinkage orthogonal test

Considering the soil thickness (A), water content (B) and time (C) as three factors in the orthogonal test group, a three factor and three-level orthogonal test was designed. With the change of site depth, there are certain differences in water content (Fig. 2), in particular, there are puddles in the middle of the site, and the water content of the soil is high, which has reached the liquid limit. Under the existing water content, the site has cracked. Obviously, we need to explore the whole process of site cracking. Therefore, the water content variables in the orthogonal test are 35% liquid limit water content, 20% plastic limit water content and their mean values. Due to the long day and high average temperature in summer, the site is subject to dry shrinkage, and the surface weathering, pulverization and peeling are serious. To explore the development of the damage depth of the earthen soil under the dry shrinkage condition, the soil layers at 1cm, 3cm and 5cm were considered in this test. In the preliminary exploratory test, it is found that the drying shrinkage time has a certain impact on the cracking of soil samples, but it is not a decisive factor. Therefore, in the dry shrinkage test, the water loss of the Zhouqiao site within 12h, 24h and 36h was primarily considered when exploring the influence of soil thickness, moisture content and simulation time on the properties of the earthen soil. The sample number and test scheme are shown in Table 2. Each group included three parallel samples for a total of 27 samples.

**Table 2:** Dry shrinkage orthogonal test scheme.

Test group	Variable		
	Soil thickness (A)	Moisture content (B)	Time (C)
A_1_B_1_C_1_	1 cm	20%	12 h
A_1_B_2_C_2_	1 cm	28%	24 h
A_1_B_3_C_3_	1 cm	35%	36 h
A_2_B_2_C_3_	3 cm	28%	36 h
A_2_B_3_C_1_	3 cm	35%	12 h
A_2_B_1_C_2_	3 cm	20%	24 h
A_3_B_3_C_2_	5 cm	35%	24 h
A_3_B_1_C_3_	5 cm	20%	36 h
A_3_B_2_C_1_	5 cm	28%	12 h

**Figure 3:**
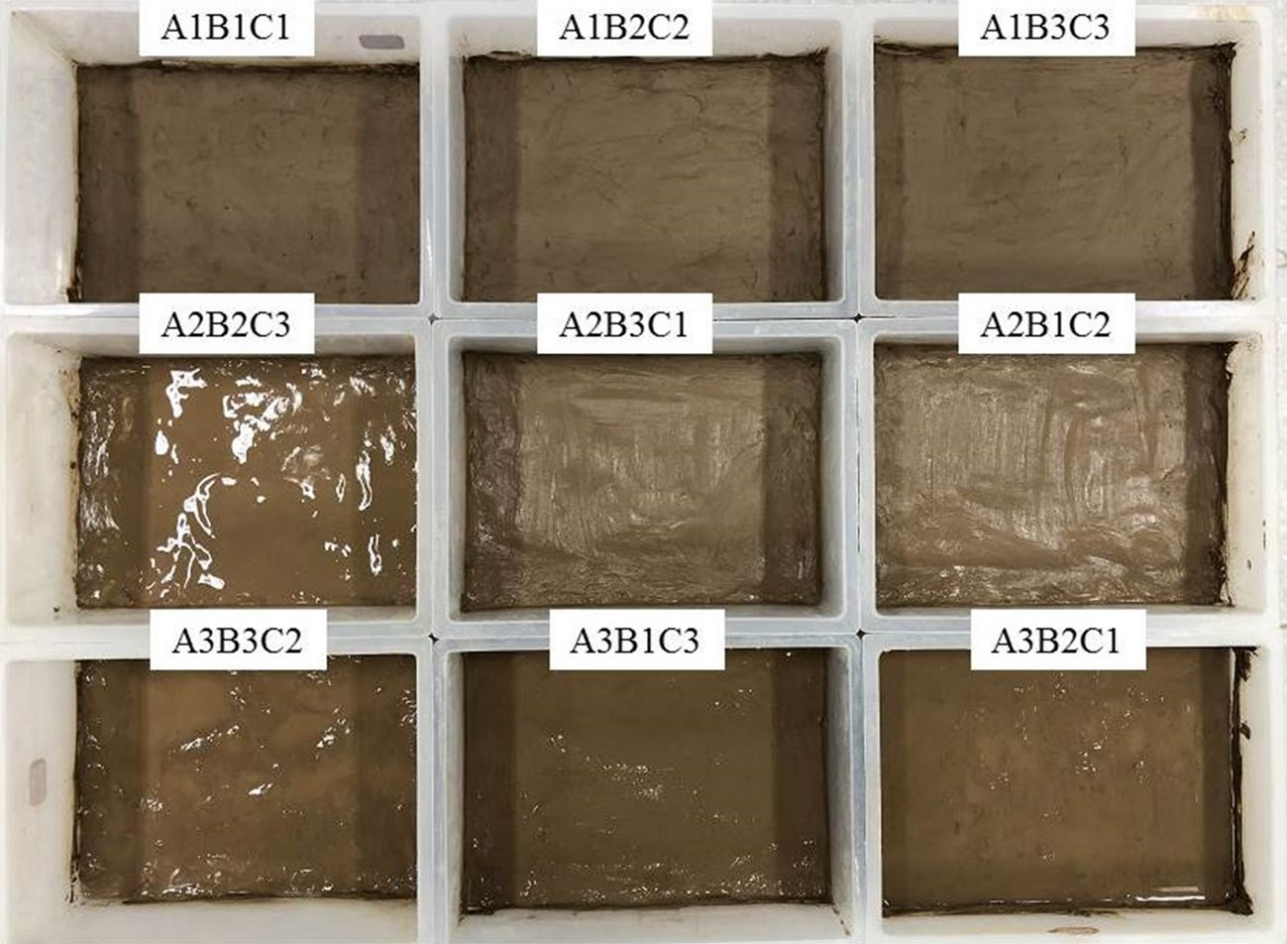
Samples and moulds.

The operational steps of the dry shrinkage orthogonal test were as follows: ① put the soil sample to be used into the oven to dry to the balance weight; ② make samples in the mould of length × ride × width = 27 cm × 17 cm × 10 cm according to the test scheme in Table 2; and ③ put the soil sample, which was cured for 24h, into a constant temperature and humidity test chamber (model KD-2P-80) for the dry shrinkage test (temperature 27 ℃, humidity 70%). The sample and die are shown in Fig. 3.

#### Freeze-thaw cycle orthogonal test

**Figure 4:**
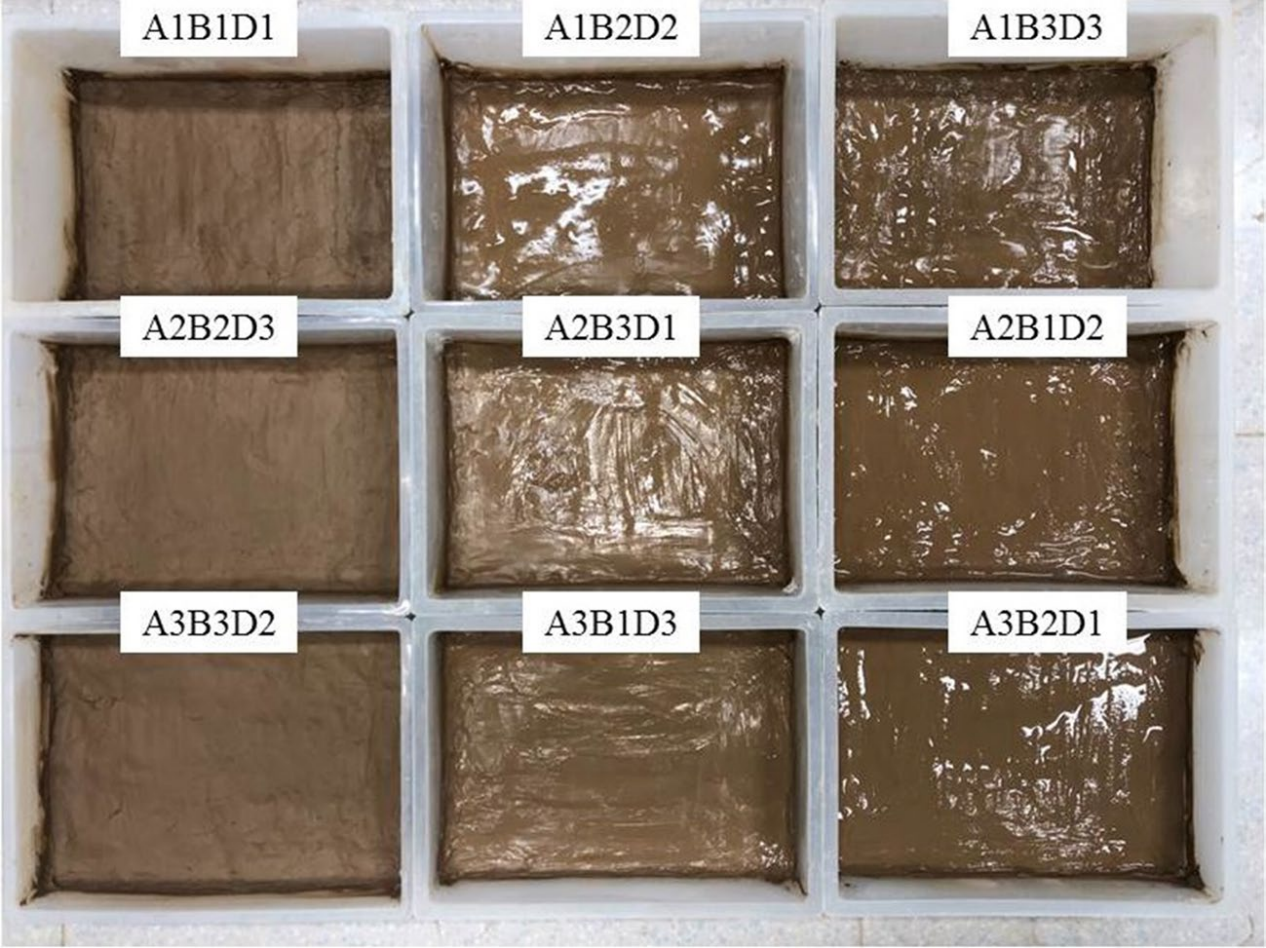
Samples and moulds.

Considering soil thickness (A), water content (B) and cycle times (D) as three test variables in the orthogonal test, a three factor and three-level orthogonal test was designed^30–33^. The sample number and test scheme are shown in Table 3. Each group includes 3 parallel samples for a total of 27 samples. The sample and mould are shown in Fig. 4.

**Table 3:** Freeze thaw orthogonal test scheme.

Test group	Variable		
	Soil thickness (A)	Moisture content (B)	Number of cycles (D)
A_1_B_1_D_1_	1 cm	20%	10 times
A_1_B_2_D_2_	1 cm	28%	20 times
A_1_B_3_D_3_	1 cm	35%	30 times
A_2_B_2_D_3_	3 cm	28%	30 times
A_2_B_3_D_1_	3 cm	35%	10 times
A_2_B_1_D_2_	3 cm	20%	20 times
A_3_B_3_D_2_	5 cm	35%	20 times
A_3_B_1_D_3_	5 cm	20%	30 times
A_3_B_2_D_1_	5 cm	28%	10 times

**Figure 5:**
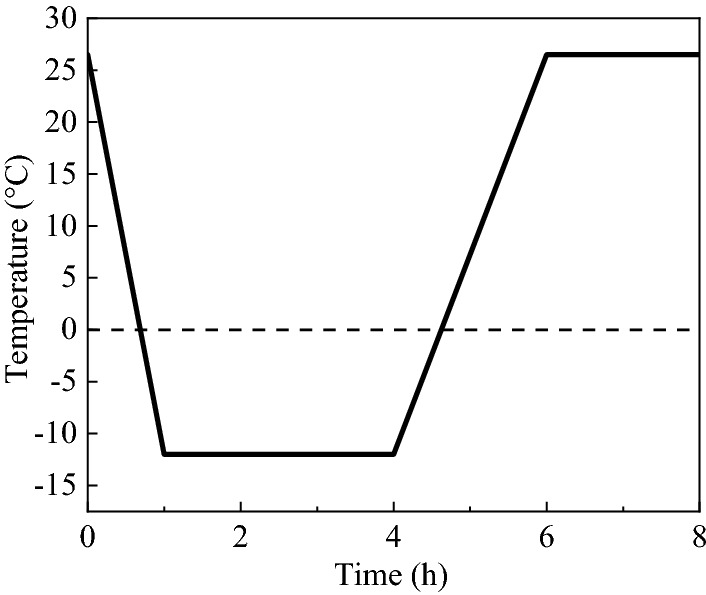
Single cycle condition.

The operational steps of the freeze-thaw cycle orthogonal test are as follows: ① put the soil sample to be used into the oven to dry to the balance weight; ② make samples in the mould of length × ride × width = 27 cm × 17 cm × 10 cm according to the test scheme in Table 3; and ③ put the samples, which were cured for 24h, into a constant temperature and humidity test box (model KD-2P-80), and conduct the test according to the freeze-thaw orthogonal test scheme in Table 3. The single cycle conditions of the freeze-thaw cycle orthogonal test are shown in Fig. 5.

### Surface strength test

To quantify the effects of drying shrinkage and freeze-thaw on the damage and mechanical property degradation of site soil samples, the surface strength of the soil samples was measured using an Adberg HLD hand-operated push-pull meter^34^. The test steps are as follows: place the soil sample on the bench of the push-pull meter, shake the handle to lower the probe with a penetration area of 28.07 mm^2^ to contact the sample surface, and clear the reading and start recording data. Select the test crack edge, sample edge and middle of each sample in turn, and calculate the surface strength using Eq. (1).

1 $$P = \frac{F}{S \times 1000}$$

where *P* is the surface strength of the sample, kPa; *F* is the pressure on the sample, N; and *S* is the surface area of push-pull meter probe, mm^2^.

### Image processing

#### Fracture rate image processing

**Figure 6:**
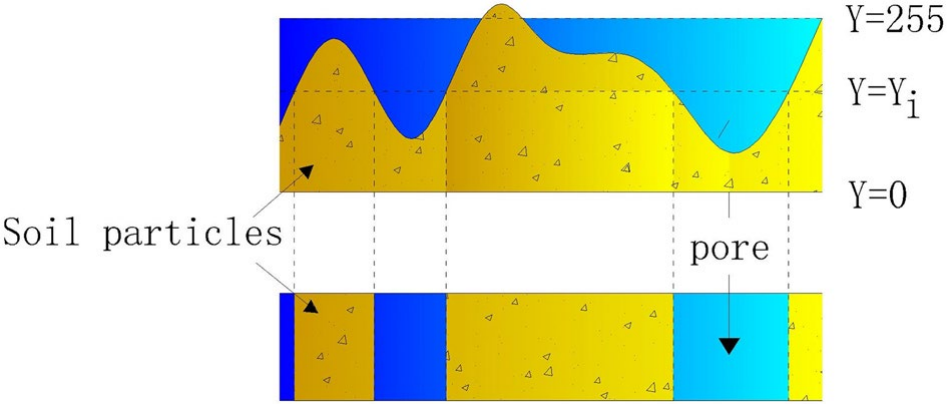
Threshold slice processing method for 3D image reconstruction.

Due to the limitation of shooting conditions in the laboratory, the degree of image recognition of the cracks is not high. It is also necessary to further process and analyse the photos of the soil samples after the dry shrinkage orthogonal test and freeze-thaw cycle test. To more intuitively demonstrate the path law of fissure development of the site soil samples and based on the threshold slice processing method of three-dimensional image reconstruction (Fig. 6). The meanings of each letter in Fig. 6 are: ① *Y* = 255 is the maximum value of image threshold, 255; ② *Y* = 0 is the minimum value of image threshold, 0; ③ *Y* = *Y*_*i*_ is the threshold corresponding to the ith pixel, and 255 > *i* > 0. The three-dimensional reconstruction of the test image was carried out using the image processing software. The original test image was binarized by the Image-Pro Plus (IPP) software, and the fracture morphological features were extracted at the same time^35^. The image processing procedure consisted of counting the number of crack pixels in the binary image, calculating the crack area of different samples, and finally, quantifying the structural damage degree of the soil samples using the crack rate (Eq. (2))^36^.

2 $$R_{{\text{n}}} = \frac{{A_{{\text{c,n}}} }}{{A_{{\text{n}}} }} \times 100\%$$

where *R*_n_ is the fracture rate, %; *A*_c,n_ is the pixel coverage area of the crack, cm^2^; and *A*_n_ is the total pixel coverage area of the image, cm^2^.

#### Surface intensity image processing

**Figure 7:**
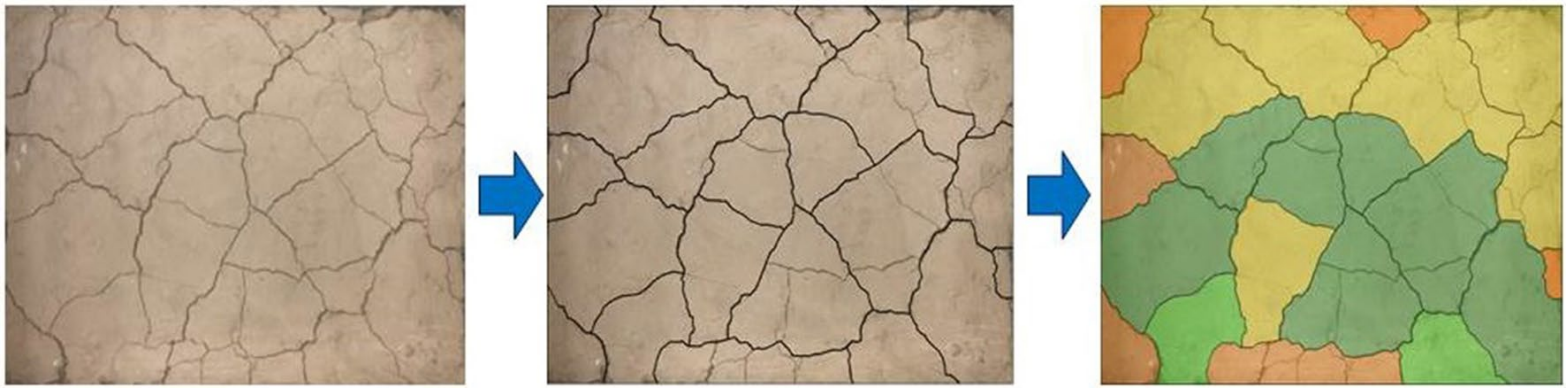
Image processing flow.

In the surface strength test, each crack area on the sample surface is tested, but there are too many test data to show better. In order to more clearly express the distribution of soil sample surface strength *P*, this paper divides the surface strength into 8 intervals, each interval shall be expressed in different colors, and the division rules are shown in Table 4. Firstly, this paper imported the captured soil sample pictures into the AutoCAD software to draw the crack path. The surface strength values of the different areas were then divided into different colours (70% transparency). The image processing flow is shown in Fig. 7. The IPP software was used to calculate area statistics for areas with different colours to obtain the proportion of the value of the corresponding surface strength interval, and the product of the area ratio and the median value of the surface strength interval was then used to measure the change in the sample surface strength under different working conditions. Finally, the overall surface strength of the corresponding sample was calculated using Eq. (3).

3 $$P = \frac{{(100 \times S_{{{\text{Orange}}}} + 300 \times S_{{{\text{Yellow}}}} + 500 \times S_{{{\text{Green}}}} + 700 \times S_{{{\text{Dark}}\,{\text{green}}}} + 900 \times S_{{{\text{Cyan}}}} + 1100 \times S_{{{\text{Blue}}}} + 1300 \times S_{{{\text{Navy}}\,{\text{blue}}}} + 1500 \times S_{{{\text{Magenta}}}} )}}{{S_{All} }} \times 100\%$$

where *P* is the surface strength, kPa and *S* is the area occupied by different surface strength values, cm^2^.

**Table 4:**
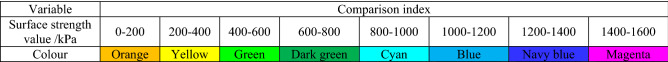
Image partition rules.

## Results

### Analysis of the dry shrinkage orthogonal test results

**Figure 8:**
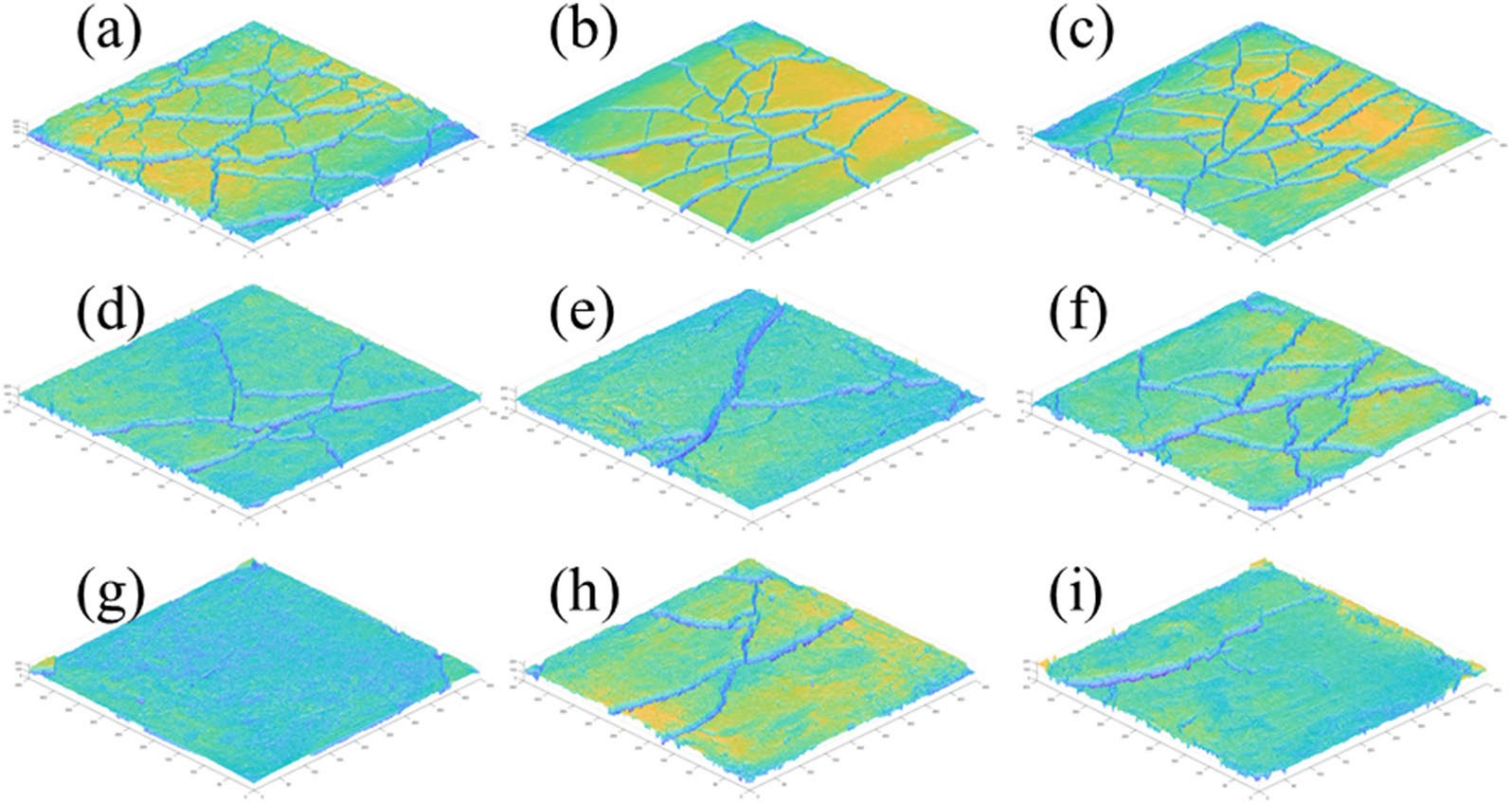
Pictures of dry shrinkage orthogonal test results. (**a**) A_1_B_1_C_1_, (**b**) A_1_B_2_C_2_, (**c**) A_1_B_3_C_3_, (**d**) A_2_B_2_C_3_, (**e**) A_2_B_3_C_1_, (**f**) A_2_B_1_C_2_, (**g**) A_3_B_3_C_2_, (**h**) A_3_B_1_C_3_, (**i**) A_3_B_2_C_1_.

Figure 8 shows the results of the dry shrinkage orthogonal test. As shown in Fig. 8, in the nine schemes of the orthogonal test, the soil samples have different degrees of cracking. The cracking of the soil samples in group A_1_B_1_C_1_ is the most obvious and the cracking of the soil samples in group A_3_B_3_C_2_ is the least obvious. Table 5 shows the fracture rate results of the dry shrinkage orthogonal test. According to Table 5, the crack rate order in this test is A_1_B_1_C_1_>A_1_B_2_C_2_>A_2_B_3_C_1_>A_1_B_3_C_3_>A_2_B_1_C_2_>A_3_B_1_C_3_>A_2_B_2_C_3_>A_3_B_2_C_1_>A_3_B_3_C_2_. Moreover, the crack rate of the A_1_B_1_C_1_ group is much higher than that of the other eight groups. In this group, the crack rate reached 8%, which is 0.8% higher than the second A_1_B_2_C_2_ group and 6.9% higher than the lowest A_3_B_3_C_2_ group. This result shows that the effects of different factors on the cracking of site soil can be observed from the orthogonal test, and the crack rate of the samples from the A_1_B_1_C_1_, A_1_B_2_C_2_ and A_1_B_3_C_3_ groups is generally higher than that of the other six groups. Additionally, the macro performance shows that the crack network is staggered. The crack rate of groups A_3_B_3_C_2_, A_3_B_1_C_3_ and A_3_B_2_C_1_ is the lowest, and it is nearly 60% lower than that of the other six groups.

**Table 5:** Dry shrinkage orthogonal test results.

Sample number	Fracture rate/%	Surface strength/kPa	Sample number	Fracture rate/%	Surface strength/kPa	Sample number	Fracture rate/%	Surface strength/kPa
A_1_B_1_C_1_	8.0	455.81	A_1_B_2_C_2_	7.2	921.54	A_1_B_3_C_3_	6.0	846.90
A_2_B_2_C_3_	3.2	989.14	A_2_B_3_C_1_	6.9	1048.10	A_2_B_1_C_2_	5.9	492.19
A_3_B_3_C_2_	1.1	1279.49	A_3_B_1_C_3_	3.3	1082.52	A_3_B_2_C_1_	2.1	1051.13

Combined with the sample cracking picture in Fig. 8 and the crack rate results in Table 5, it can be concluded that the cracking degree of the site soil layer is inversely proportional to the thickness of the soil layer. The thinner the soil layer, the more the soil layer cracks first, resulting in a staggered crack network. With an increase in the thickness of the site’s soil layer, only a single and coarse main crack appears in the soil layer. Through observation during the test, it was found that the cracking degree of the earthen soil layer is also inversely proportional to the initial water content of the soil layer, and the soil sample with low initial water content is the first to crack. The cracks of soil samples with large initial moisture content develop slowly. For example, the cracks in the A_3_B_3_C_2_ group soil samples exist at the corners of the sample.

**Figure 9:**
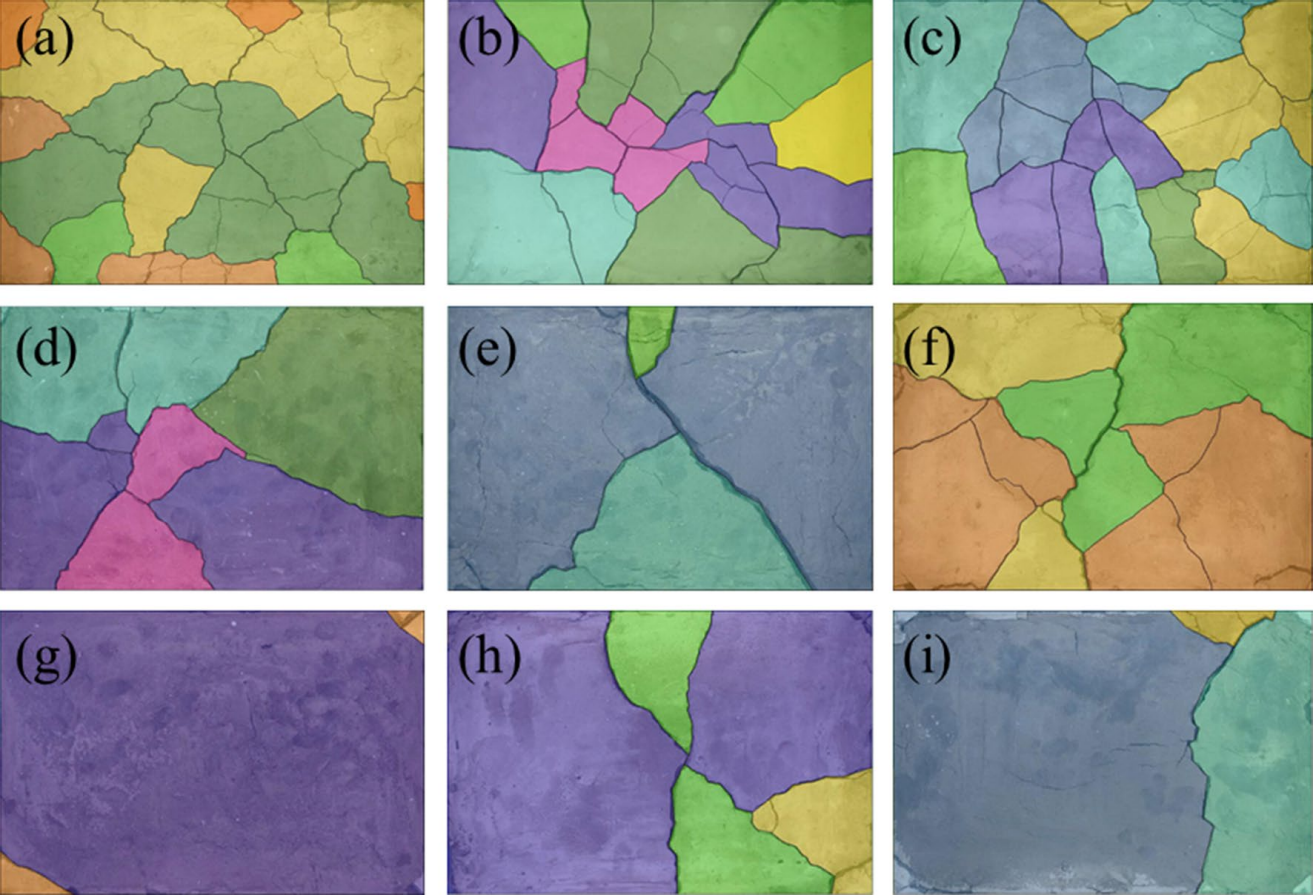
Nominal surface strength results of dry shrinkage orthogonal test. (**a**) A_1_B_1_C_1_, (**b**) A_1_B_2_C_2_, (**c**) A_1_B_3_C_3_, (**d**) A_2_B_2_C_3_, (**e**) A_2_B_3_C_1_, (**f**) A_2_B_1_C_2_, (**g**) A_3_B_3_C_2_, (**h**) A_3_B_1_C_3_, (**i**) A_3_B_2_C_1_.

Figure 9 shows the surface strength results of the dry shrinkage orthogonal test. According to the surface strength results in Table 4, the surface strength order from this test is A_3_B_3_C_2_>A_3_B_1_C_3_>A_2_B_3_C_1_>A_3_B_2_C_1_>A_2_B_2_C_3_>A_1_B_2_C_2_>A_1_B_3_C_3_>A_2_B_1_C_2_>A_1_B_1_C_1_. The results show that surface strength is inversely proportional to the law of crack rate, that is, the greater the crack rate, the smaller the surface strength of the sample. In addition, it shows that with the development of cracks, the surface strength of the site soil samples weakens. For example, the crack rate of the samples in the A_3_B_3_C_2_, A_3_B_1_C_3_ and A_3_B_2_C_1_ groups is generally lower than that of the other six groups, while the surface strength of the soil samples is higher than that of the other six groups.

As shown in Fig. 9 of the manuscript, the surface strength around the soil sample is generally lower than that in the middle of the soil sample. For example, in Fig. 9a, the surface strength around the soil sample is 0–200 kPa, and the surface strength in the middle of the soil sample is 600–800 kPa; in Fig. 9d, the surface strength around the soil sample is 800–1000 kPa, and the surface strength in the middle of the soil sample is 1400–1600 kPa. Moreover, the crack rate of group A_1_B_1_C_1_ in Fig. 9a is 8.0%, which is higher than that of group A_2_B_2_C_3_ in Fig. 9d. The results also show the following relationship between surface strength and crack distribution: ① As the number of cracks increases with an increase in drying time, the integrity of the soil sample structure is destroyed, resulting in the reduction of each block area and smaller surface strength; ② The surface strength around the soil sample is low when there are increased cracks, and the internal strength of the sample is high when there are fewer cracks; ③ The smaller the crack development of the sample, the more complete the surface is. Therefore, in the safety evaluation and restoration of earthen sites, attention should be paid to the influence of surface micro cracks and through cracks on their mechanical properties.

The range *R*-value reflects the influence of an experimental factor on the result index. The greater the *R*-value, the greater the influence of this test factor on the result index. It can be seen from Table 6 that the *R*-value order of the three test factors on the fracture rate is A>B>C, and the *R*-value of test factor A is much greater than that of the latter two test factors, indicating that within the selected factor level range, the primary factor affecting the increase in the fracture rate is A. The order of the *R* values of the three test factors on the surface strength is B>A>C, and the *R*-value of test factor B is much greater than that of the other two test factors, indicating that the primary factor affecting the increase in the surface strength is B within the selected factor level. As the soil site is affected by the storage environment, water can only be lost from the site’s surface, and the surface soil is supplemented with water inside the soil sample. Therefore, the thinner the soil sample, the easier it is to cause water loss cracking and the lower the surface strength. The other two factors were also analysed using this method. When the soil sample is 1cm thick with 20% moisture content, after 12h, earthen soil cracking and surface strength reduction is most likely to occur.

**Table 6:** Range analysis of dry shrinkage orthogonal test results.

*K* value	Index					
	Fracture rate			Surface strength		
	Factor					
	Soil thickness	Moisture content	Time	Soil thickness	Moisture content	Time
	A	B	C	A	B	C
*K*_**1**_	21.2	17.2	17	2224.3	1582.1	2555.0
*K*_**2**_	16	12.5	14.2	2529.4	2961.8	2693.2
*K*_**3**_	6.5	14	14	2964.8	3174.5	3174.5
*k*_1_	7.1	5.7	5.7	741.4	527.4	851.7
*k*_2_	5.3	4.2	4.7	843.1	987.3	897.7
*k*_3_	2.2	4.7	4.7	988.3	1058.2	1058.2
*R*	14.7	4.7	3	740.5	1592.4	619.5
Primary and secondary order	A > B > C			B > A > C		

### Analysis of freeze–thaw orthogonal test results

**Figure 10:**
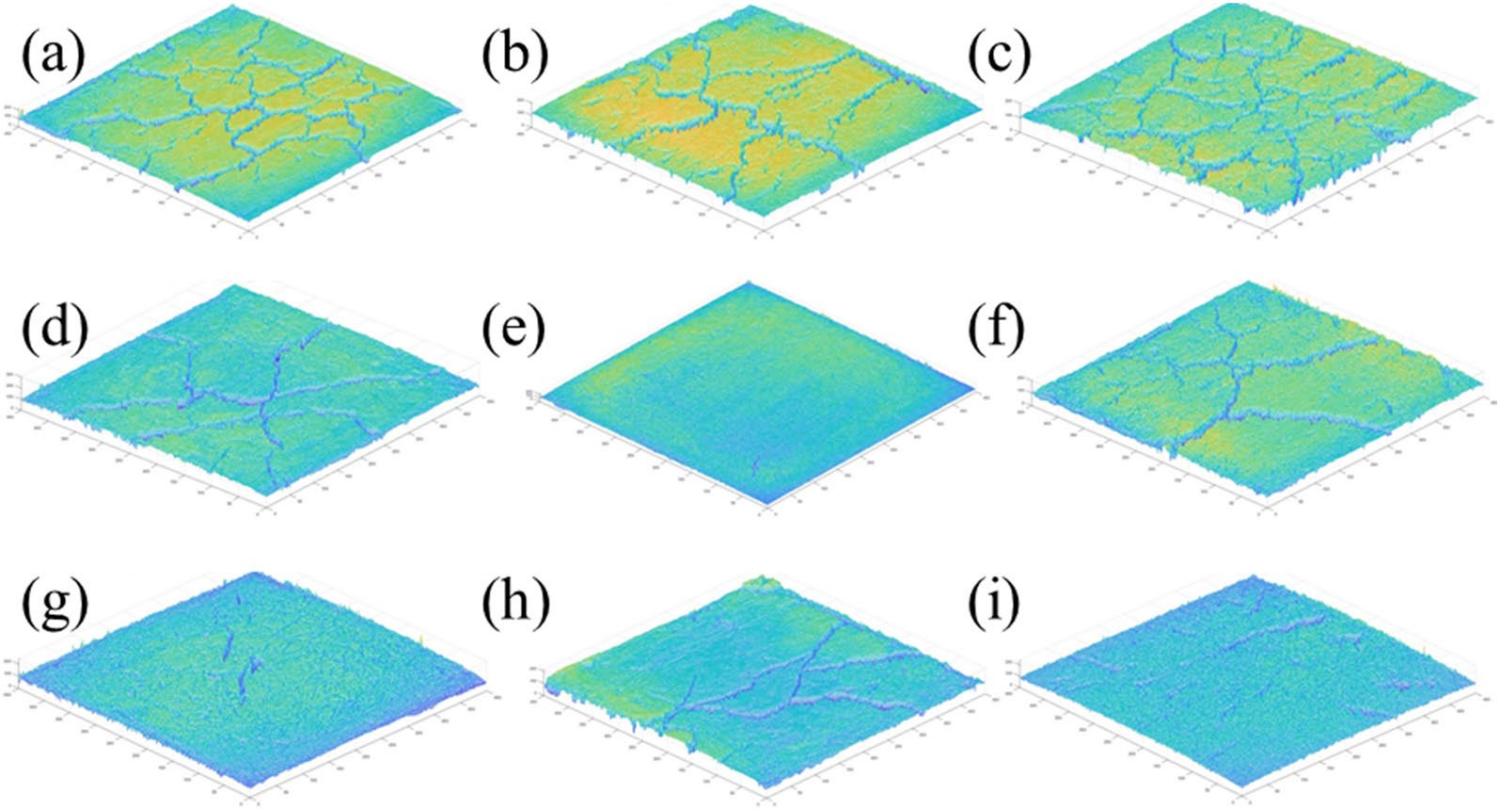
Pictures of freeze–thaw orthogonal test results. (**a**) A_1_B_1_D_1_, (**b**) A_1_B_2_D_2_, (**c**) A_1_B_3_D_3_, (**d**) A_2_B_2_D_3_, (**e**) A_2_B_3_D_1_, (**f**) A_2_B_1_D_2_, (**g**) A_3_B_3_D_2_, (**h**) A_3_B_1_D_3_, (**i**) A_3_B_2_D_1_.

Figure 10 shows the results of the freeze-thaw orthogonal test. As shown in Fig. 10, in the nine schemes of the orthogonal test, the soil samples have different degrees of cracking, of which the cracking of the soil samples in group A_1_B_1_D_1_ is the most obvious and the cracking of the soil samples in group A_2_B_3_D_1_ is the least obvious. Table 7 shows the fracture rate results of the freeze-thaw orthogonal test. According to Table 7, the crack rate order in this test is A_1_B_1_D_1_>A_2_B_1_D_2_>A_1_B_2_D_2_>A_2_B_2_D_3_>A_1_B_3_D_3_>A_3_B_1_D_3_>A_3_B_3_D_2_>A_3_B_2_D_1_>A_2_B_3_D_1_. The crack rate of the A_1_B_1_D_1_ group is much higher than that of the other eight groups. In this group, the crack rate reached 7.8%, which was 1.6% higher than the second A_2_B_1_D_2_ group and 7.5% higher than the lowest A_2_B_3_D_1_ group.

Combined with the sample cracking picture in Fig. 10 and the crack rate results in Table 7, it can be concluded that the cracking degree of the site soil layer is affected by the initial water content. The greater the initial water content, the smaller the crack rate of the site soil sample; additionally, the time when the crack occurs is later. By recording the macro phenomenon of the soil samples after each freeze-thaw cycle, it was found that the soil samples with low initial moisture content have obvious main cracks and long crack lengths, while the soil samples with high initial moisture content have micro-cracks and wide crack distribution ranges. In the process of moisture absorption, the soil suction decreases, the mechanical properties of the soil samples weaken, the soil at the edge of the cracks collapse due to the existence of the overhead surface, and the collapsed soil sample fills in the crack and effectively heals it^37^. Therefore, it can be observed that the cracks healed after a freeze-thaw cycle "reappear" at the original crack position in the subsequent cycle, and more micro-cracks develop near the original crack.

**Figure 11:**
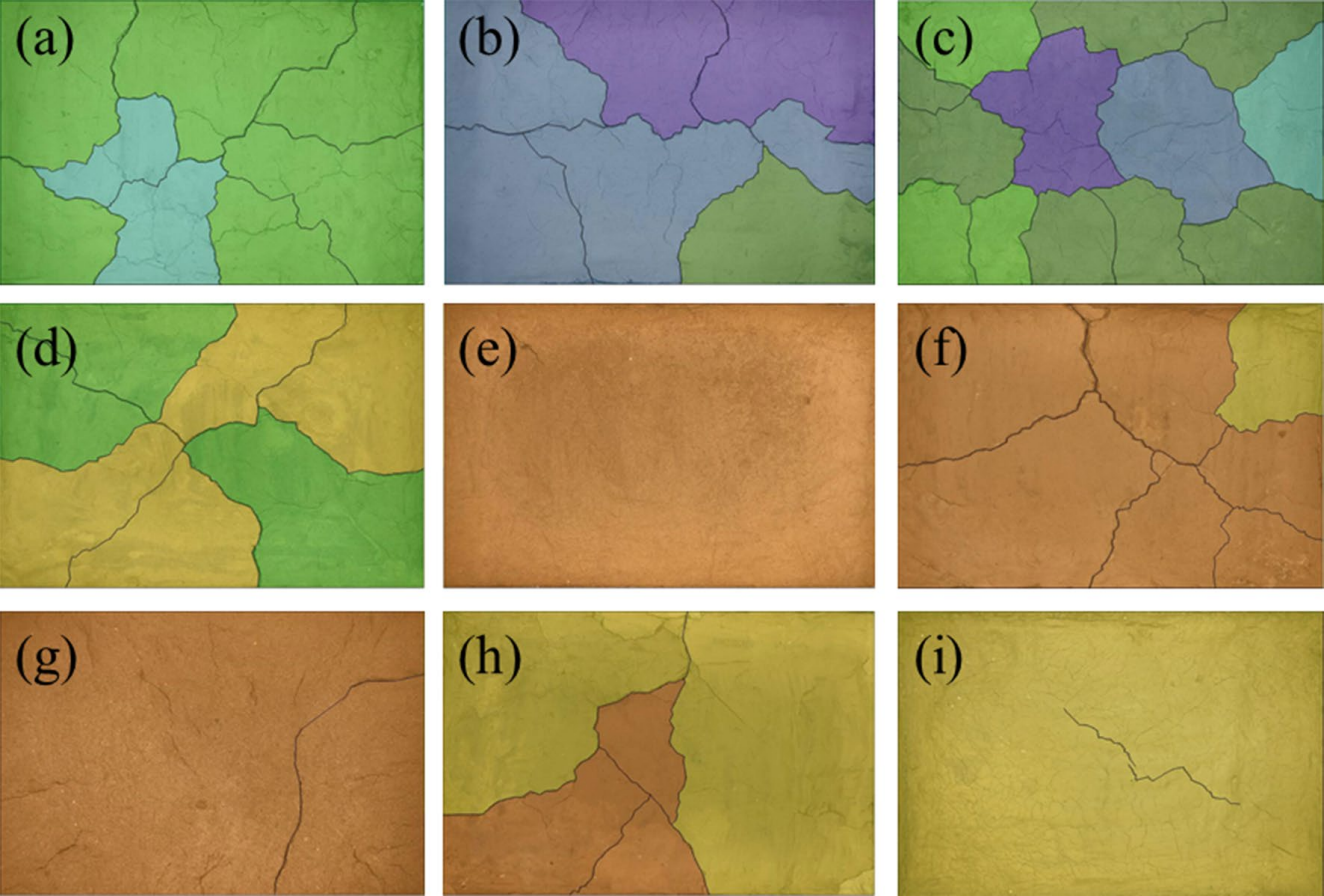
Nominal surface strength results of freeze–thaw orthogonal test. (**a**) A_1_B_1_D_1_, (**b**) A_1_B_2_D_2_, (**c**) A_1_B_3_D_3_, (**d**) A_2_B_2_D_3_, (**e**) A_2_B_3_D_1_, (**f**) A_2_B_1_D_2_, (**g**) A_3_B_3_D_2_, (**h**) A_3_B_1_D_3_, (**i**) A_3_B_2_D_1_.

Figure 11 shows the surface strength results of the freeze-thaw orthogonal test. Table 7 shows the surface strength results. According to Table 7, the surface strength order from the freeze-thaw orthogonal test is A_1_B_2_D_2_>A_1_B_3_D_3_>A_1_B_1_D_1_>A_2_B_2_D_3_>A_3_B_2_D_1_>A_2_B_1_D_2_>A_3_B_1_D_3_>A_2_B_3_D_1_=A_3_B_3_D_2_. The surface strength results are directly proportional to the crack rate, that is, the greater the crack rate, the greater the surface strength of the sample. The primary reason for this phenomenon is that under the action of freeze-thaw cycling, with an increase in soil layer thickness, the water loss rate of the earthen soil sample is slower. When the test was completed, the sample was still wet and the crack rate therefore developed slowly. The thickness of the test samples from the A_1_B_1_D_1_, A_1_B_2_D_2_ and A_1_B_3_D_3_ groups was 1cm, which is thinner than that of the other six groups. From the crack rate results, it can be concluded that the surface layer has lost water and cracks. However, due to the water loss on the surface, the soil samples agglomerate under the action of cohesion, resulting in an increase in surface strength.

**Table 7:** Results of freeze–thaw orthogonal test.

Sample number	Fracture rate/%	Surface strength/kPa	Sample number	Fracture rate/%	Surface strength/kPa	Sample number	Fracture rate/%	Surface strength/kPa
A_1_B_1_D_1_	7.8	607.34	A_1_B_2_D_2_	5.8	1100.75	A_1_B_3_D_3_	4.3	888.65
A_2_B_2_D_3_	5.1	392.31	A_2_B_3_D_1_	0.3	100.00	A_2_B_1_D_2_	6.2	281.94
A_3_B_3_D_2_	2.2	100.00	A_3_B_1_D_3_	3.3	246.78	A_3_B_2_D_1_	1.1	300.00

According to the freeze-thaw test results, the surface strength and crack distribution are different from the dry shrinkage orthogonal test: ① affected by the thickness of the soil layer, the thicker the soil layer and the more water in the soil, the smaller the sample’s crack development and the lower the surface strength; ② the strength near the soil sample is low and there are many cracks. The internal strength of the sample is high and there are few cracks.

It can be seen from Table 8 that the *R*-value order of the three test factors of the fracture rate is B>D>A; the *R*-value of test factor B is much greater than that of the last two test factors, indicating that within the selected factor level range, the primary factor affecting the increase in the fracture rate is B. The *R*-value order of the three test factors of the surface strength is A>B>D, and the *R*-value of test factor A is much greater than that of the other two test factors, indicating that within the selected factor level, the primary factor affecting the increase in surface strength is A. The primary mechanism of soil freezing and thawing is the freezing and thawing of the water in the soil pores. During this process, the surface soil sample first experiences frost heaving, and then with the continuation of low temperatures, the frost heaving depth deepens. Then, the water molecules in the soil are adsorbed on the surface of the soil particles under the action of soil particle surface force. When the soil temperature is lower than the freezing temperature of free water, the water in the soil begins to freeze. At this stage, the frozen water fills in the pores of the soil sample to inhibit cracking of the soil sample. With an increase in temperature, the frozen water gradually changes from solid to liquid. The pores between the soil particles are affected by surface tension, resulting in secondary cracking and finally, an increase in the erodibility of the soil sample structure, which makes the sample more vulnerable to erosion^38^.

**Table 8:** Range analysis of freeze–thaw orthogonal test results.

*K* value	Index					
	Fracture rate			Surface strength		
	Factor					
	Soil thickness	Moisture content	Number of cycles	Soil thickness	Moisture content	Number of cycles
	A	B	D	A	B	D
*K*_**1**_	17.9	17.3	9.2	2596.7	1136.1	1007.3
*K*_**2**_	11.6	12	14.2	774.3	1793.1	1482.7
*K*_**3**_	6.6	6.8	6.8	646.8	1088.7	1088.7
*k*_1_	6.0	5.8	3.1	865.6	378.7	335.8
*k*_2_	3.9	4.0	4.7	258.1	597.7	494.2
*k*_3_	2.2	2.3	2.3	215.6	362.9	362.9
*R*	6.3	10.5	7.4	1950.0	657.0	475.4
Primary and secondary order	B > D > A			A > B > D		

In the freezing phase, the soil on the surface layer gradually forms ice with the internal water, which continuously fills the pores of the surface layer, generating expansion forces that promote the closure of existing microfractures and produce a thin ice layer^39,40^. During the melting phase, the thin ice layer on the surface of the soil sample continuously melts, the crystalline water changes from a solid to a liquid state and gradually evaporates along with the higher external ambient temperature, the surface tension between the particles becomes greater and the tensile stress between the particles increases, promoting the development of microfractures. The internal temperature of the specimen during the melting phase is lower than the ambient temperature relative to the ambient temperature specimen, causing greater stress and deformation discordance between the internal and external surfaces of the specimen due to the excessive difference between the internal and external materials and the temperature difference, causing the soil to thaw settlement^41^. Therefore, the macroscopic performance can be summarized as follows: the crack rate of soil samples in the melting stage is greater than that of those in the freezing stage^42^.

In conclusion, under the freeze-thaw condition, when the soil sample is 1cm thick with 20% moisture content, after 20 cycles, the freeze-thaw cracking is the most serious; the surface strength of the site soil decreases most significantly when samples are less than 5cm thick with 35% moisture content after 10 freeze-thaw cycles.

### Microstructure analysis

**Figure 12:**
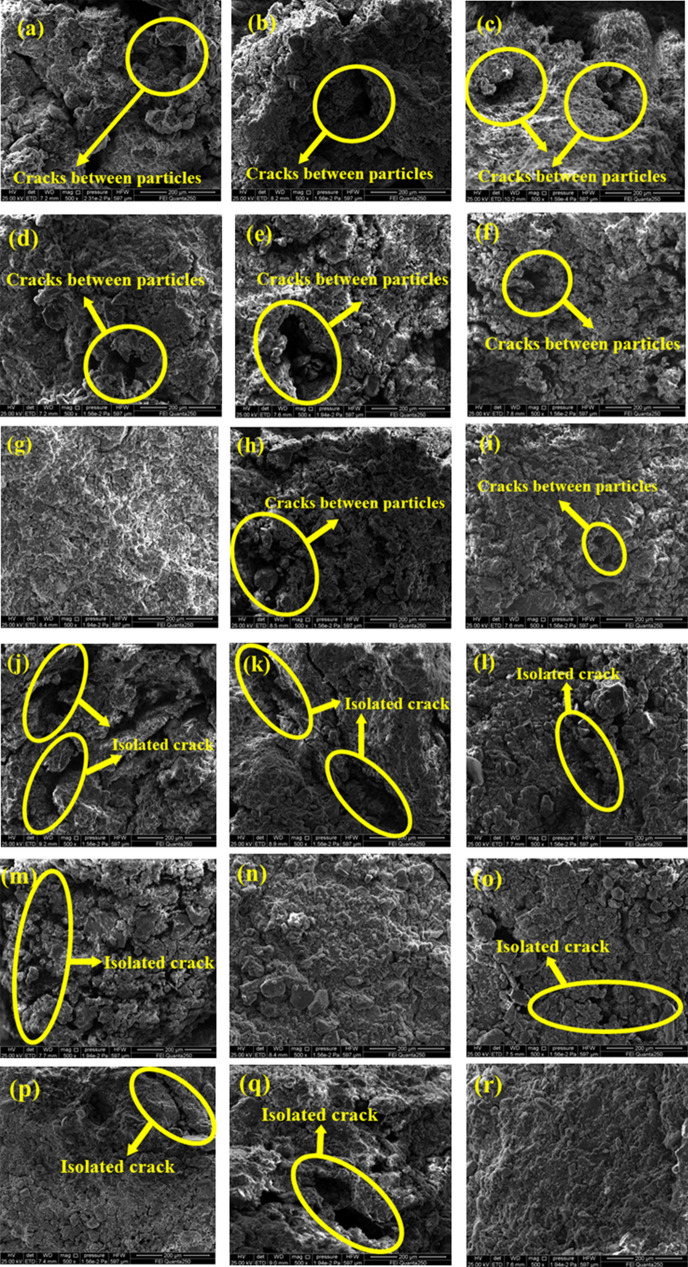
SEM results of drying shrinkage and freeze–thaw orthogonal test. (**a**) A_1_B_1_C_1_, (**b**) A_1_B_2_C_2_, (**c**) A_1_B_3_C_3_, (**d**) A_2_B_2_C_3_, (**e**) A_2_B_3_C_1_, (**f**) A_2_B_1_C_2_, (**g**) A_3_B_3_C_2_, (**h**) A_3_B_1_C_3_, (**i**) A_3_B_2_C_1_, (**j**) A_1_B_1_D_1_, (**k**) A_1_B_2_D_2_, (**l**) A_1_B_3_D_3_, (**m**) A_2_B_2_D_3_, (**n**) A_2_B_3_D_1_, (**o**) A_2_B_1_D_2_, (**p**) A_3_B_3_D_2_, (**q**) A_3_B_1_D_3_, (**r**) A_3_B_2_D_1_.

Due to space limitations, only one electron microscope from each group is listed in Fig. 12. To prevent the influence of test error on the test results during sample making, the entire geotechnical test was conducted according to the standard for geotechnical test methods (GB/T50123-2019). The manufacturing process was consistent with other test conditions except for the difference in the test dosage. Figure 12 shows the SEM results of the dry shrinkage and freeze-thaw orthogonal tests. It can be seen from Fig. 12 that the earthen soil has a bedding structure and that the basic particle units are in contact with each other. The spatial forms of the cracks between the soil samples are primarily isolated and intergranular cracks^43^; the length of the isolated cracks is large, the distribution is discontinuous and generally exhibits an irregular strip distribution, and the intergranular cracks generally exhibit circular or elliptical distributions. The SEM results of the dry shrinkage orthogonal test primarily focus on the distribution of intergranular cracks, and the SEM results of the freeze-thaw cycle orthogonal test primarily focus on the distribution of isolated cracks, as shown in Fig. 12.

IPP software with 500 times magnification was used to process the SEM images^44^, and the pore area ratio of the SEM images was obtained (Table 9). Through the analysis of Table 9, it can be found that the magnitude of the crack area ratio shown by the microstructure of the dry shrinkage and freeze-thaw orthogonal test samples is basically consistent with the magnitude of the macro crack rate. According to Fig. 12 and Table 9, due to the initial damage in the soil sample, the damaged cracks provide evaporation and infiltration paths for water, and the soil around the pores experience dry shrinkage first. With the migration of water, the cracks gradually deepen and finally, they form a "Pit" on the soil sample’s macro scale. This phenomenon is consistent with the conclusion proposed by Tang^45^ that the existence of "miscellaneous points" in the soil samples leads to disharmony between local shrinkage deformation and the surrounding areas during the drying process, thus inducing the formation of cracks.

**Table 9:** SEM image fracture area ratio.

Sample number	A_1_B_1_C_1_	A_1_B_2_C_2_	A_1_B_3_C_3_	A_2_B_2_C_3_	A_2_B_3_C_1_	A_2_B_1_C_2_	A_3_B_3_C_2_	A_3_B_1_C_3_	A_3_B_2_C_1_
Fracture area ratio	0.34	0.31	0.25	0.20	0.31	0.23	0.05	0.13	0.09

Sample number	A_1_B_1_D_1_	A_1_B_2_D_2_	A_1_B_3_D_3_	A_2_B_2_D_3_	A_2_B_3_D_1_	A_2_B_1_D_2_	A_3_B_3_D_2_	A_3_B_1_D_3_	A_3_B_2_D_1_
Fracture area ratio	0.40	0.38	0.41	0.33	0.15	0.56	0.29	0.38	0.07

**Figure 13:**
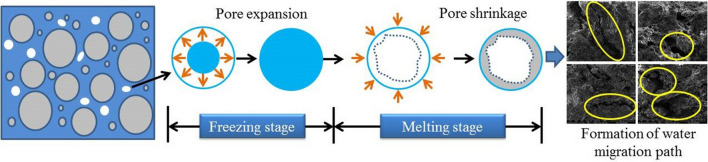
Schematic diagram of freeze–thaw cycle process.

Figure 13 is a schematic diagram of the freeze-thaw cycle process. According to analysis of Figs. 12, 13 and Table 9, when the ambient temperature is low, the temperature gradient and water potential gradient in the soil are large, and a large amount of water migrates to the surface of the soil sample and crystallizes and expands in the pores. Severe water vapor migration greatly reduces the relative humidity of the pores in the soil sample, causing the pores in the soil sample to first expand and then contract, which results in an irregular water migration path. The macro performance of water migration to the surface of soil sample in the form of water vapor causes the water content of the surface of the soil sample to increase greatly; this phenomenon causes the surface of the soil sample to approach a saturated state, which explains the relationship between thicker soil samples and lower surface strength during the orthogonal test. The thinner the soil sample, the lower the internal water content and the evaporation of water at the sample surface is. It is therefore difficult for the surface of the soil sample to approach the saturated state, which leads to cracking.

### Micro characteristic analysis under dry shrinkage condition

**Figure 14:**
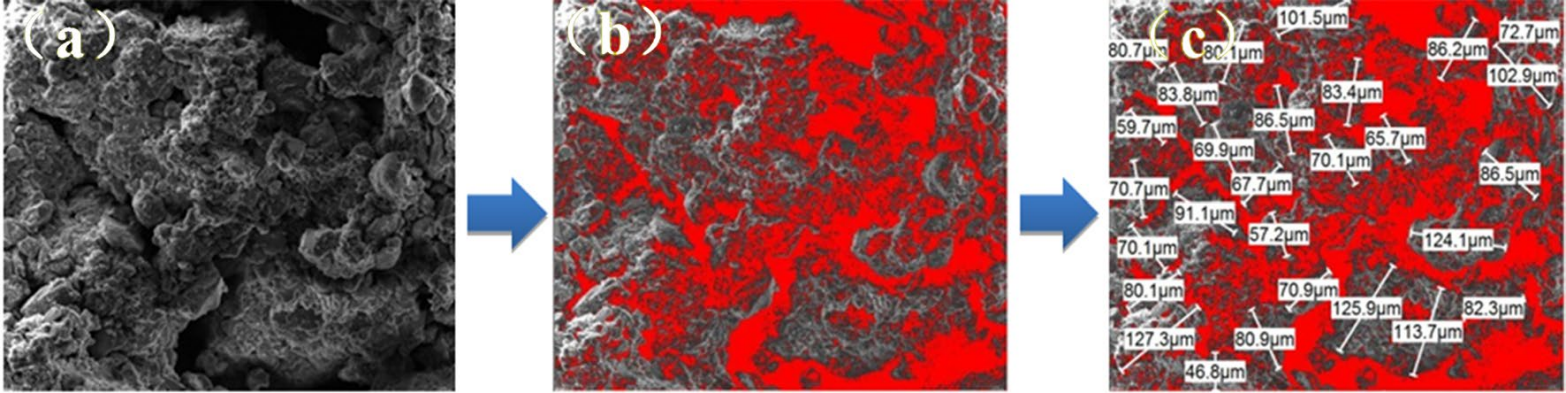
Image processing flow. (**a**) SEM image. (**b**) IPP software processing results. (**c**) Smileview software processing results.

The SEM images of the earthen soils (Fig. 12) were processed by applying the count and measure objects function of the IPP software. In order to match the distribution of the processed images to the real soil sample microstructure, the area of the pore space was determined based on the results of the mercury-pressure test porosity. The SEM images of the soils from the Zhouqiao site (Fig. 12) were then labelled for soil particle size by SmileView software and the mean particle size was calculated for each SEM image. The mean results (0.075cm and 0.078cm) were in general agreement with the mean grain size of the soil samples (0.074cm), demonstrating the feasibility of the method. The results are shown in Tables 10 and 11, and the processing flow is shown in Fig. 14.

**Table 10:** Average particle size of SEM image of dry shrinkage orthogonal test sample.

Test group	A_1_B_1_C_1_	A_1_B_2_C_2_	A_1_B_3_C_3_	A_2_B_2_C_3_	A_2_B_3_C_1_	A_2_B_1_C_2_	A_3_B_3_C_2_	A_3_B_1_C_3_	A_3_B_2_C_1_	Mean value
Average particle size/mm	0.084	0.081	0.076	0.071	0.078	0.074	0.069	0.070	0.069	0.075

**Table 11:** Average particle size of SEM image of freeze–thaw orthogonal test sample.

Test group	A_1_B_1_C_1_	A_1_B_2_C_2_	A_1_B_3_C_3_	A_2_B_2_C_3_	A_2_B_3_C_1_	A_2_B_1_C_2_	A_3_B_3_C_2_	A_3_B_1_C_3_	A_3_B_2_C_1_	Mean value
Average particle size/mm	0.086	0.083	0.079	0.081	0.071	0.084	0.074	0.077	0.074	0.078

**Figure 15:**
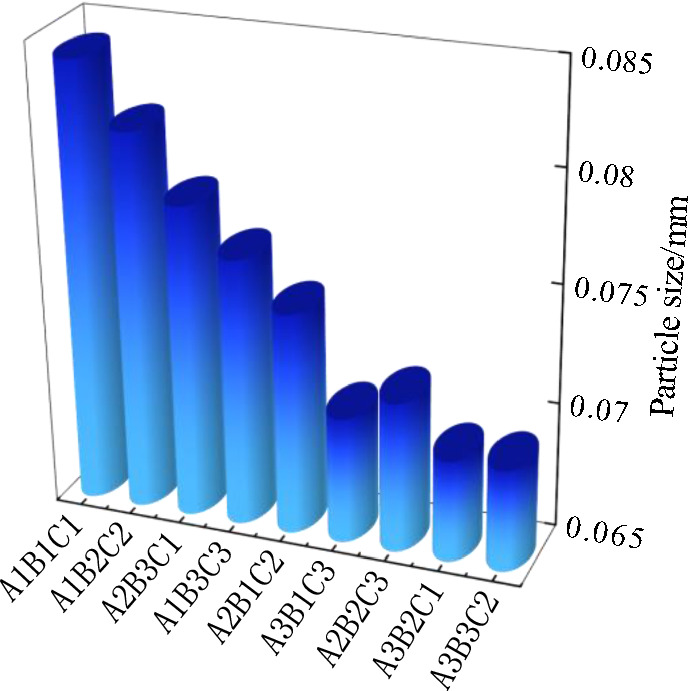
Graph of particle size versus fractality.

**Figure 16:**
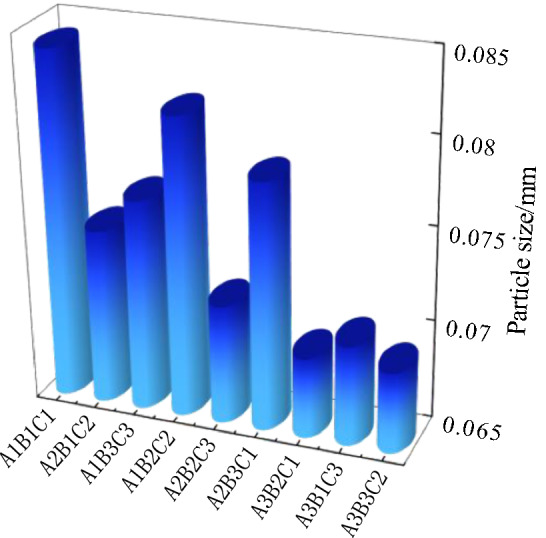
Graph of particle size versus surface strength.

**Figure 17:**
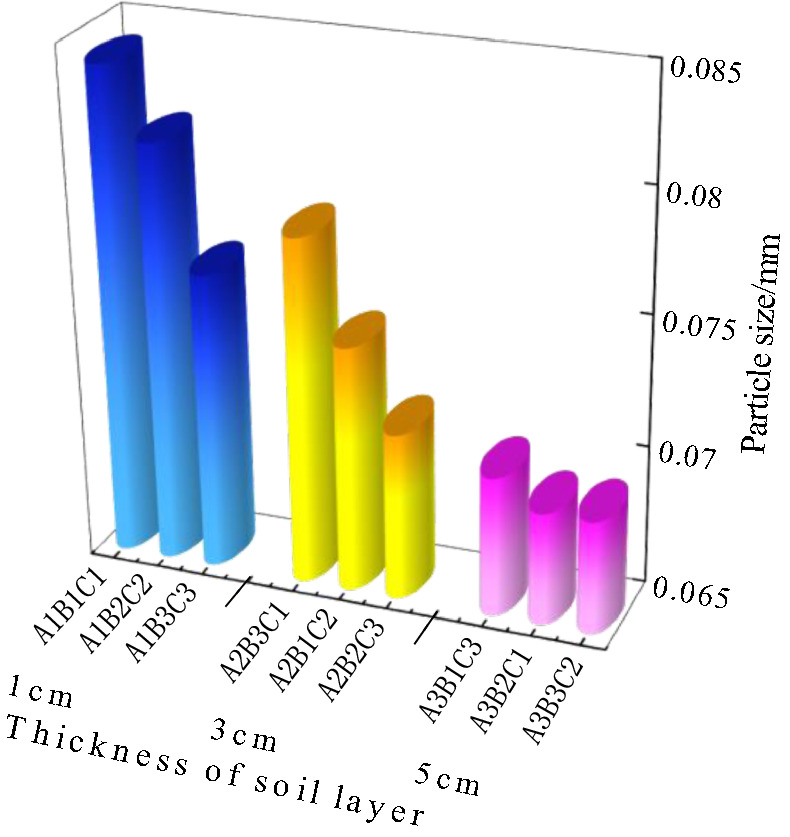
Graph of particle size versus soil thickness.

Based on the data analysis in Tables 5 and 10, the relationship between particle size and crack rate (Fig. 15), the relationship between particle size and surface strength (Fig. 16) and the relationship between particle size and soil thickness (Fig. 17) can be obtained, respectively. According to the dry shrinkage orthogonal test, the order of crack rate is A_1_B_1_C_1_>A_1_B_2_C_2_>A_2_B_3_C_1_>A_1_B_3_C_3_>A_2_B_1_C_2_>A_3_B_1_C_3_>A_2_B_2_C_3_>A_3_B_2_C_1_>A_3_B_3_C_2_. As can be seen from Fig. 15, the particle size of the particle agglomerates within the soil sample decreases as the fractality decreases. Based on the mechanism of the surface tension F, as the particle size of the soil sample increases, the water holding capacity of the soil sample becomes weaker and the inter-particle microscopic forces gradually increase, eventually leading to fissures on the surface of the soil sample. As can be seen from Fig. 16, the surface strength and the particle size of the particle agglomerates inside the soil sample generally show a negative correlation, which is due to the fact that the microstructure composition of the smaller particle size is denser and the internal skeleton is more uniformly stressed, resulting in an increase in strength. As can be seen from Fig. 17, the particle size of the particle agglomerates showed some variation after the dry shrinkage test for specimens with different soil thicknesses. The particle size of the agglomerates of the 1cm thick specimen was the largest, with an average value of 0.080cm, while the particle size of the 3cm thick specimen was close to the average particle size of 0.074cm and the smallest particle size of the 5cm thick specimen, with an average value of 0.069cm. This is due to the internal water content of the soil layer. This is due to the fact that the water content inside the soil layer is small and the evaporated water cannot be replenished in time. The contraction force between the particles increases, which leads to the formation of agglomerates and cracks on the surface of the specimen.

### Micro characteristic analysis under freeze–thaw condition

**Figure 18:**
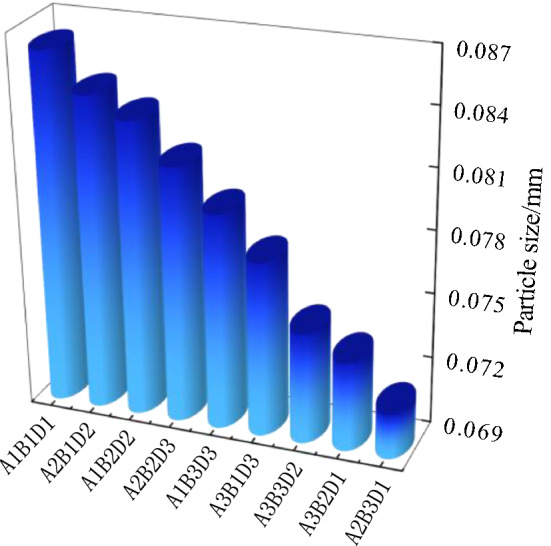
Graph of particle size versus fractality.

**Figure 19:**
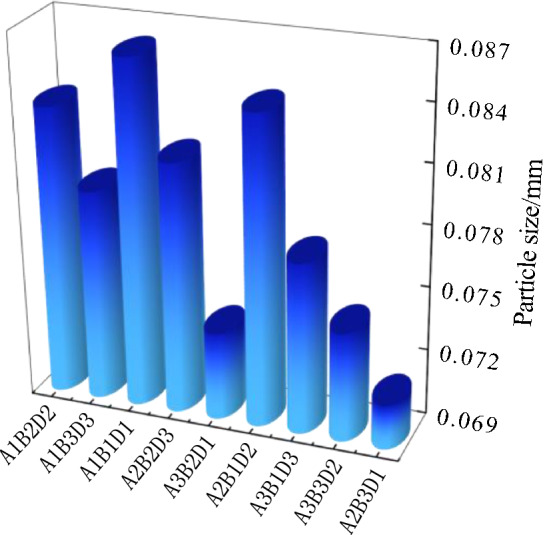
Graph of particle size versus surface strength.

**Figure 20:**
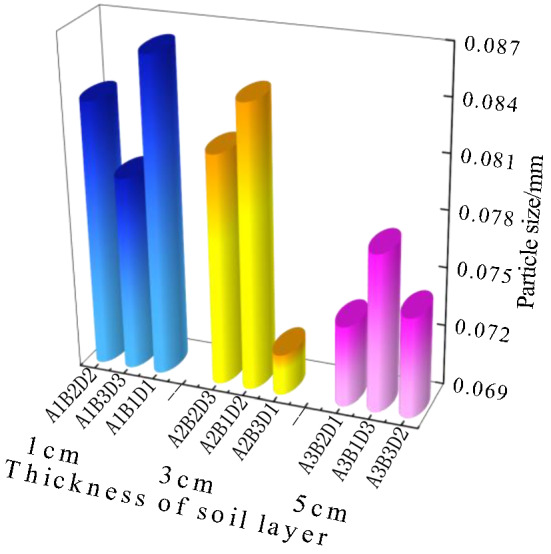
Graph of particle size versus soil thickness.

Based on the data analysis in Tables 7 and 11, the relationship between particle size and crack rate (Fig. 18), the relationship between particle size and surface strength (Fig. 19) and the relationship between particle size and soil thickness (Fig. 20) can be obtained, respectively. The freeze-thaw orthogonal test shows that the order of the fractality is A_1_B_1_D_1_>A_2_B_1_D_2_>A_1_B_2_D_2_>A_2_B_2_D_3_>A_1_B_3_D_3_>A_3_B_1_D_3_>A_3_B_3_D_2_>A_3_B_2_D_1_>A_2_B_3_D_1_. As can be seen from Fig. 18, the particle size of the particle agglomerates inside the soil sample decreases as the fractality decreases, which is due to the weaker water-holding capacity of the larger particle sizes. This is due to the weaker water holding capacity of the larger size soil samples and the effect of water migration on the microscopic particle distribution, which causes the cracking to be more pronounced in the larger size soil samples. As can be seen from Fig. 19, the particle size of the particle agglomerates within the soil sample generally tends to decrease as the surface strength increases. Due to the small particle size, moisture is not easily crystallised within the smaller pores under freeze-thaw conditions, and moisture moves more towards the surface layer, resulting in a low surface strength. From Fig. 20, the particle size of the particle agglomerates of the 1cm thick specimen is the largest, with an average value of 0.083 cm, while the particle size of the 3cm thick specimen is close to the average particle size of 0.078 cm of the site soil sample, and the particle size of the 5cm thick specimen is the smallest, with an average value of 0.075 cm. The greater the thickness, the higher the internal water content, the greater the difference between the internal temperature of the specimen and the ambient temperature, and the constant migration of water to the surface layer of the specimen, the greater the change in the microstructural composition of the soil sample. For example, the average particle size of the agglomerates in a 5cm thick sample is 0.075 cm, which is in line with the average particle size of the soil samples at the site (0.074 cm), while thinner soils are more susceptible to freeze-thaw and crystallisation on the surface of the sample, resulting in an increase in the size of the agglomerates.

## Inter-particle microscopic forces

### Inter-particle microscopic shrinkage forces under dry shrinkage conditions

The cracking of the soil sample is mainly caused by the shrinkage of the soil sample due to evaporative water loss. In order to be able to discriminate the cracking of soil samples, to determine whether the soil samples will crack under certain stress conditions, and to predict the depth and width of fracture development in the soil samples, etc. Scholars at home and abroad have proposed some theoretical models, and the mechanical theoretical models for cracking in soil samples are divided into four main categories^46^: tensile damage models, fracture mechanical theoretical models, body change models and stress path analysis models.

The tension damage models are the most commonly used class of soil cracking models. The basic criterion for this type of model is that cracking will occur when the tensile stress inside the soil exceeds its tensile strength. Fracture mechanics is analysed from an energy point of view and the parameters involved are difficult to quantify in practice, so most of the research related to this type of model is currently at the theoretical stage. The body-variation model and the stress-path analysis model only analyse the cracking of soil samples as a whole, and it is difficult to find out the specific values of parameters such as the length, width and depth of the fracture in practice.

Based on the above analysis, the cracking behaviour was found to be directly related to the shrinkage properties of the soil sample. At the macroscopic scale, the soil sample stress-strain relationship is the most influential factor on the physical properties of the soil, while at the microscopic scale, surface tension has the greatest influence on the generation of cracks in the soil sample. Li et al.^47^ found through their study that moisture between soil sample particles larger than 10 nm exists mainly in the form of liquid bridges and that moisture within the pores can have an effect on the stability of the soil sample.

Therefore, in this paper, the theoretical equation for the relationship between microcapillary inter-pore forces and water content is established by using different surface tensions as gradient influencing factors, while keeping the macroscopic physical properties of the soil sample unchanged. Ultimately, the determination of whether the soil sample is fractured or not is made through the definition of a tension damage class model.

**Figure 21:**
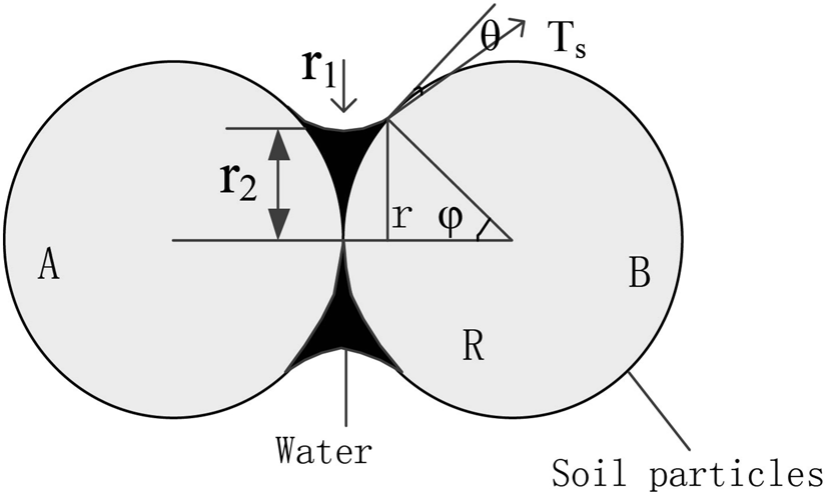
Sketch of the forces on the soil particles.

**Figure 22:**
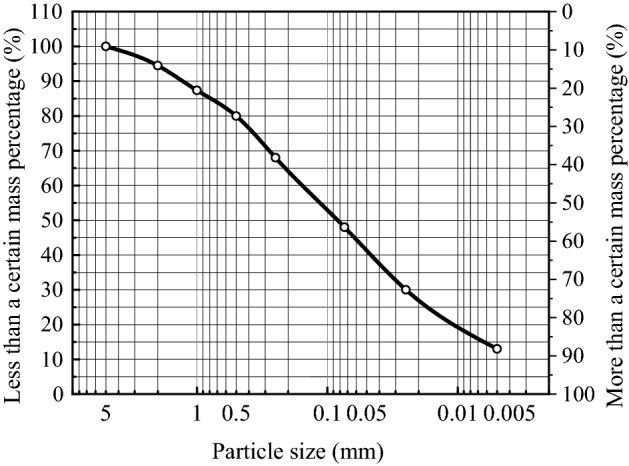
Particle gradation curves for eathen soils.

**Figure 23:**
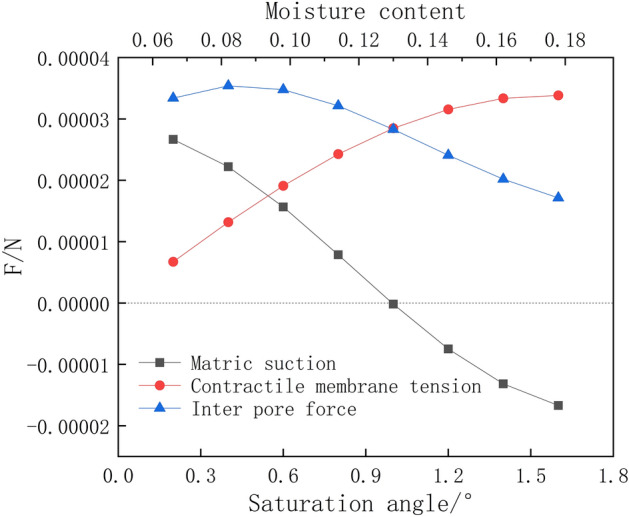
Capillary inter-pore forces versus water content.

The suction forces contributing to the interaction between unsaturated soil particles include five types of suction forces: intrinsic structural suction, variable structural suction, effective matrix suction (pore gas pressure, pore water pressure), wet suction and traction. Only the effective matrix suction, the wet suction and the variable structural suction vary with water content, soil structure and environmental chemistry, etc. The sum of these three suction forces can be referred to as the generalised suction. Figure 21 shows a sketch of the forces on the soil particles. There is a bending moon surface water film between the soil particles, and the capillary forces caused by the bending moon surface cause a surface tension *F* between the soil particles. As can be seen from Fig. 21, the surface tension *F* is composed of matrix suction *F*_*c*_ and suction *T*_*s*,_ (Eq. (8)). The average particle size of soil particles in this paper is 0.074 mm (Fig. 22), and the surface tension coefficient of water under normal pressure α The relationship between (N/m) and temperature T (℃) can be expressed as α = 7.564 × 10^−2^–1.4 × 10^−4 ^T, and the contact angle is 10°. Ultimately, by fitting Eq. (8), a graph of the variation of the microcapillary inter-pore force versus water content is obtained (Fig. 23). From the definition of the tensile damage class model, it is clear that when the microscopic inter-pore force is greater than the cohesive force of the soil sample (Eq. (9)), the soil sample will sprout fractures.

The matrix suction *F*_*c*_ is

4 $$F_{{\text{c}}} = \pi {\text{r}}^{2} (u_{a} - u_{w} ) = \pi \alpha {\text{r}}^{2} (1/r_{1} - 1/r_{2} )$$

5 $$r_{1} = R(1 - \cos \varphi )/\cos (\varphi + \theta )$$

6 $$r_{2} = R(\sin (\varphi + \theta ) + \cos \varphi - \sin \theta - 1)/\cos (\varphi + \theta )$$

where *r*_1_ and *r*_2_ are the two curvature radii of the opposite meniscus; *u*_*α*_ is pore gas pressure; *u*_*ω*_ is pore water pressure; *θ* is the contact angle between water and solid surface; *φ* is the saturation angle; *α* is the surface tension coefficient.

The shrink film tension *T*_*s*_ is

7 $$T_{{\text{s}}} = 2\pi {\text{r}}\alpha = 2\pi {\text{R}}\alpha \sin \varphi$$

From Eqs. (4) and (7), the surface tension *F* is

8 $$\begin{aligned} F & = F_{{\text{C}}} + F_{{\text{S}}} \\ & = \pi \alpha ({\text{R}}\sin \varphi )^{2} (1/r_{1} - 1/r_{2} ) + 2\pi {\text{R}}\alpha \sin \varphi \\ & = \pi {\text{R}}\alpha \sin \varphi \left\{ {\sin \varphi \left( {\frac{\cos (\varphi + \theta )}{{1 - \cos \varphi }} - \frac{\cos (\varphi + \theta )}{{\sin (\varphi + \theta ) + \cos \varphi - \sin \theta - 1}}} \right) + 2} \right\} \\ \end{aligned}$$

9 $$F > [c]$$

where *F* is the surface tension and *c* is the cohesive force.

### Inter-particle microscopic forces under freeze–thaw conditions

Based on the capillary theory, taking the pressure difference formed by the meniscus at the ice water interface as the main driving force of water migration and combined with the thermodynamic equilibrium equation of ice water interface. Everett^48^ established the capillary theory. That is, the first frost heaving theory (Eq. (10)). Subsequent tests have proved that this formula is applicable to soil samples composed of dispersed particles^49^. With the proposal of young Laplace Eq. (11), the change relationship of water in the process of migration is supplemented.

10 $$P_{i} - P_{w} = \frac{{\rho_{w} L}}{{T_{m} }}(T_{m} - T)$$

where *P*_*i*_ is ice pressure; *P*_*w*_ is water pressure; *ρ*_*w*_ is the density of water; *L* is the latent heat of hydrothermal phase transition; *T*_*m*_ is the freezing temperature of water; *T* is the temperature.

11 $$P_{i} - P_{w} = \frac{{2\gamma_{iw} }}{r}$$

where *γ*_*iw*_ is the surface tension coefficient of ice water interface; *r* is the radius of water passing through the pores.

If the radius *r* of water passing through the pores is greater than the effective radius *r*_*p*_, water cannot migrate through the pores^50^. The famous Gibbs-Thomson equation can be obtained by simultaneous Eqs. (10) and (11). Gibbs-Thomson equation solves this problem by introducing the temperature *T*_*p*_ during water migration, that is, when the temperature *T* < *T*_*p*_, the water migration stops. When the temperature *T* = *T*_*p*_, the maximum frost heaving pressure *P*_*imax*_ can be obtained.

12 $$P_{imax} = P_{w} + \frac{{2\gamma_{iw} }}{{r_{p} }}$$

When the maximum frost heaving pressure *P*_*imax*_ is greater than the cohesion of the soil sample (Eq. (13)), cracks will sprout in the soil sample.

13 $$P_{imax} > [c]$$

where *P*_*imax*_ is the maximum frost heaving pressure and *c* is the cohesion.

## Discussion

**Figure 24:**
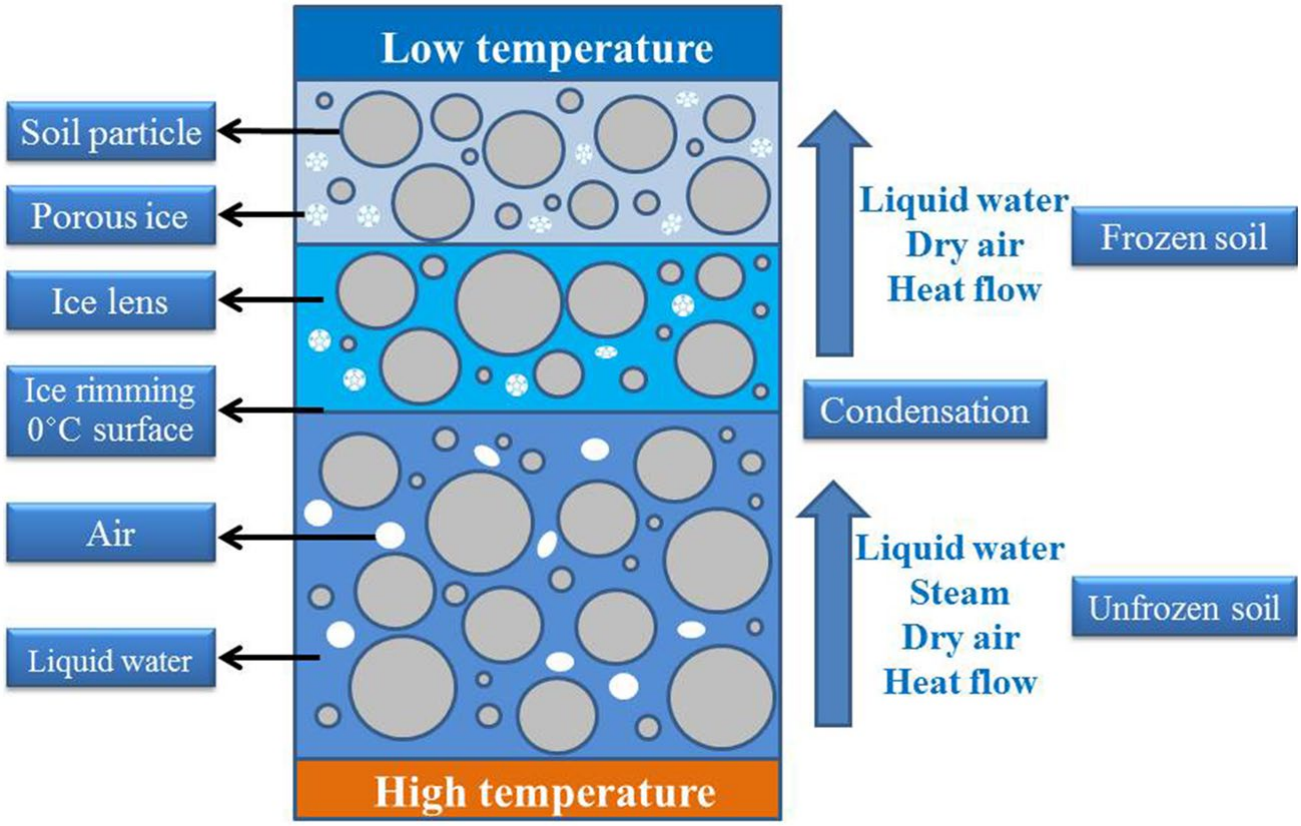
Schematic diagram of capillary frost heaving model of earthen soil.

Frost heaving of fissure area ratio soil is related to the phenomenon that when the temperature is lower than 0 ℃, the water freezing volume between the soil particles expands, causing the relative movement of those particles. This process results in frost heaving deformation or frost heaving force on the soil^50^. Xue et al.^51^ proved through experimentation that frost heaving in soil is caused by the migration of liquid water. The schematic diagram of the capillary frost heaving model of earthen soil is shown in Fig. 24, which shows the movement of heat, liquid water and air in site soil. When a temperature gradient is formed in the soil, under the action of the water potential gradient, unfrozen liquid water and steam migrate along the direction of temperature reduction^40^. Wet air moves to the freezing front (where the temperature in the soil is equal to the freezing point) because it has the same potential energy gradient as liquid water. In addition, due to the low temperature, the steam condenses at the edge of the ice^52^, which means that only liquid water and dry air can be transferred to the freezing area. With further migration of liquid water, a thin ice layer is generated on the sample’s surface. In the soil sample melting stage, the movement of heat, liquid water and air in the earthen soil is opposite to that in Fig. 24. After the pore crystalline water in the upper section of the soil sample melts, a small portion of the water evaporates through the surface while the other portion migrates to the inside of the sample through the "throat", resulting in water loss and cracking on the surface of the soil sample.

**Figure 25:**
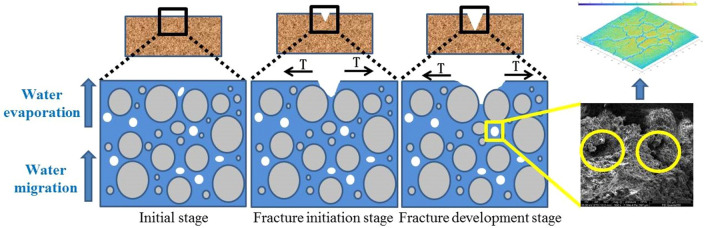
Schematic diagram of dry shrinkage cracking process.

Through studying the mechanism of dry shrinkage cracking of earthen sites, it was found that soil cracking is related to internal water loss^53^. During the process of water loss and shrinkage, the pore size between the soil particles decreases and the compactness increases. With the continuous migration of water from the interior of the soil sample to the surface, when the relative humidity of the surface soil is higher than the atmospheric relative humidity, water molecules evaporate^54^. Under the combined action of capillary pressure and surface tension^55^, cracks in the samples first appear near intergranular pores. The existence of cracks increases the soil’s weathering depth and intensifies the surface erosion^56,57^ (Fig. 25). After the formation of a fracture network, the smaller the thickness of the soil layer, the lower the water content and the lower the surface strength.

## Conclusion

In this paper, through dry shrinkage and freeze-thaw orthogonal tests on the Zhouqiao site’s earthen soil as well as range analysis, image processing technology, surface strength measurement and microstructure analysis, we comprehensively explored the effect differences of influencing factors such as soil thickness, moisture content, dry shrinkage time and freeze-thaw cycle times on the morphological characteristics of earthen soil fracture networks. The primary conclusions are as follows:

1. Under the action of dry shrinkage, the primary factors that affect the cracking and surface strength reduction of the site soil are the soil’s layer thickness and initial water content. The thinner the soil layer, the lower the water content is and the more serious the cracking is. Due to the initial damage in the soil sample, the damaged cracks provide evaporation and infiltration paths for water. With the migration of water, the cracks gradually deepen. The water evaporation near the surface of the soil sample is faster than that in the middle, resulting in low strength and additional cracks. When the soil sample is 1 cm thick with 20% moisture content, after 12 h action time, the cracking is the most serious.
2. Under the action of freezing and thawing, the primary factors that affect the cracking and surface strength reduction of the earthen soil are the initial moisture content and soil layer thickness, respectively. The thinner the soil layer thickness, the more serious the cracking is. At the beginning of the freeze-thaw cycle, the pores gradually expand and the intergranular pore wall is damaged. With an increase in the number of freeze-thaw cycles, the tensile failure of the pore edge is more obvious, and finally, a fracture network is formed. When the soil sample is 1cm thick with 20% moisture content, after 20 freeze-thaw cycles, the sample’s cracking is the most serious; when the soil sample is 5 cm thick with 35% moisture content, after 10 freeze-thaw cycles, the surface strength of the sample decreases most significantly.
3. The SEM results show that the internal cracks in soil samples have different shape characteristics according to whether they experienced dry shrinkage or freeze-thaw conditions. During the drying process, the local shrinkage deformation of the soil sample is inconsistent with the surrounding area, so the intergranular cracks generally appear under the drying shrinkage condition. The isolated cracks generally appear in the soil samples from the freeze-thaw cycle test. The different types of cracks caused by the two external environments cause damage and damage to the site soil.
4. The theoretical relationship between the microcapillary inter-pore forces and the water content was established by calculating the inter-particle microscopic contraction forces through different surface tensions as gradient influencing factors. The relationship equation for the maximum freezing pressure is obtained by the first frost and swelling theory. According to the tension failure model and the definition of the first frost heaving theory, it can be determined that when the micro pore force *F* and the maximum frost heaving pressure *P*_*Imax*_ are greater than the cohesion of the soil sample, the soil sample will germinate cracks.

## Data Availability

The data used to support the findings of this study are included within the article.
